# IDH mutations may not preclude distant, trans-tentorial spread in gliomas: a case report and review of the literature

**DOI:** 10.1186/s12957-016-0814-8

**Published:** 2016-02-24

**Authors:** Christopher S. Hong, Jason K. Hsieh, Nancy A. Edwards, Abhik Ray-Chaudhury, Kareem A. Zaghloul

**Affiliations:** Surgical Neurology Branch, National Institute of Neurological Disorders and Stroke, National Institutes of Health, 10 Center Drive, Building 10, Room 3D20, Bethesda, MD 20892 USA; The Ohio State University College of Medicine, 410 W. 10th Avenue, Columbus, OH 43210 USA; Cleveland Clinic Lerner College of Medicine, 9500 Euclid Ave./NA21, Cleveland, OH 44195 USA

**Keywords:** Cerebellum, Glioma, Isocitrate dehydrogenase, Recurrence

## Abstract

**Background:**

IDH mutations have been demonstrated to confer prolonged survival in patients suffering from gliomas, but the mechanisms underlying the improved prognosis are unclear. While some studies have attributed these observations to an enhanced sensitivity to genotoxic therapies, others have postulated that IDH-mutated gliomas exhibit less aggressive intrinsic biological behavior, including the propensity to invade distant sites. Although most gliomas recur local to the site of initial presentation, some tumors demonstrate distant recurrence, the vast majority of which involve the contralateral hemisphere. Trans-tentorial spread has been described once before, in which a supratentorial glioblastoma was reported to recur infratentorially in the cerebellum.

**Case presentation:**

We describe a patient who underwent surgical resection, followed by adjuvant radiation and temozolomide of a World Health Organization (WHO) III anaplastic astrocytoma in the right temporal lobe, exhibiting an IDH1 (R132H) mutation. Twenty-two months after surgery, he developed a second lesion, located in the right cerebellum, suspicious for recurrent tumor versus radiation necrosis. A second surgery was performed, and pathology demonstrated recurrent tumor, consistent with IDH1-mutated anaplastic astrocytoma.

**Conclusions:**

This is the first example of trans-tentorial spread in an IDH-mutated glioma, suggesting that despite improved survival, IDH mutations may not preclude gliomas from exhibiting the ability to invade distant sites of the brain.

## Background

Gliomas are malignant tumors arising from astrocytes within the brain and are classified into grades II to IV, based on histopathological criteria set forth by the World Health Organization (WHO). In recent years, mutations in isocitrate dehydrogenase 1 (IDH1) and IDH2 have been shown to characterize low-grade glioma and secondary glioblastomas [1]. Moreover, IDH mutations confer a significant survival benefit, compared to their wild type counterparts, across all WHO grades [2]. As such, routine assessment of IDH mutation status is becoming increasingly commonplace in the clinical setting.

It is well known that gliomas frequently recur despite optimal surgery and adjuvant radiation and chemotherapy. Up to 90 % of all recurrences are found within 2–3 cm from the area of initial presentation [3, 4], while recurrence involving the contralateral hemisphere occurs in up to 4 % of cases [5]. The role of IDH mutations in the propensity for distant recurrence is unclear although some evidence suggests that IDH wild-type gliomas may have a stronger predilection for such behavior [5].

In this report, we describe a patient who underwent surgical resection of a right temporal lobe WHO III IDH1-mutated anaplastic astrocytoma, followed by adjuvant radiation and temozolomide. Twenty-two months after surgery, he developed a second lesion in the right cerebellum, for which he underwent total resection. Pathology again demonstrated an IDH-mutated anaplastic astrocytoma, suggesting similar origin from the original tumor. Our patient is the first example of an IDH-mutated glioma exhibiting behavior of trans-tentorial recurrence. As such, IDH mutations, despite association with improved survival, may not preclude the ability of gliomas to recur distantly from the initial site of presentation.

## Case presentation

**Fig. 1:**
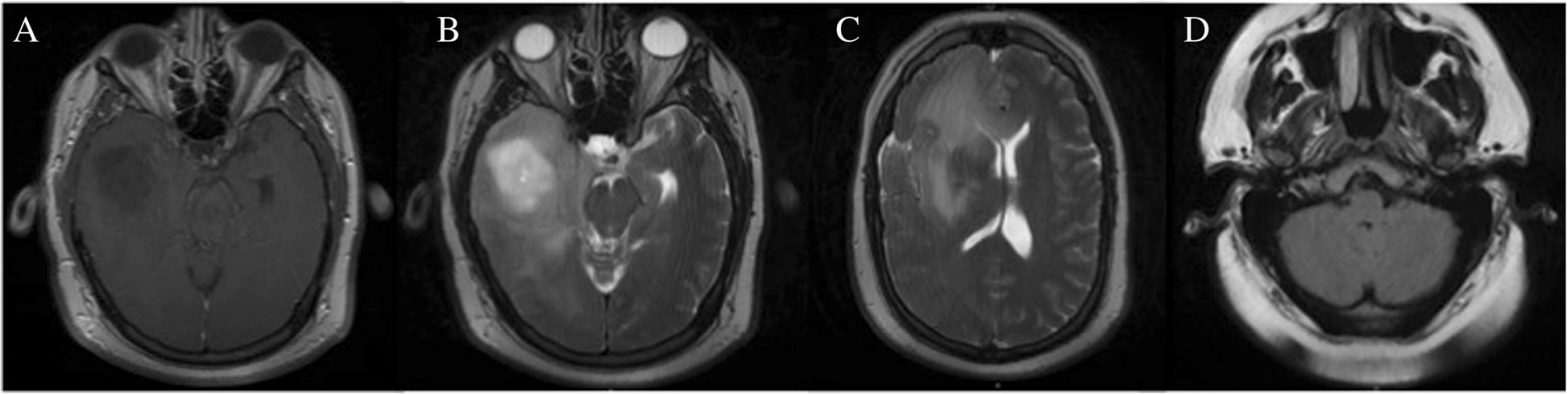
Imaging features of the initial tumor. **a** T1-weighted post-contrast and **b** T2-weighted MRI demonstrated a non-enhancing lesion in the right temporal lobe. Edema extended superiorly on T2-weighted MRI (**c**) to involve the fronto-parietal lobes and corpus callosum. **d** T2-weighted FLAIR MRI did not reveal any disease in the cerebellum

**Fig. 2:**
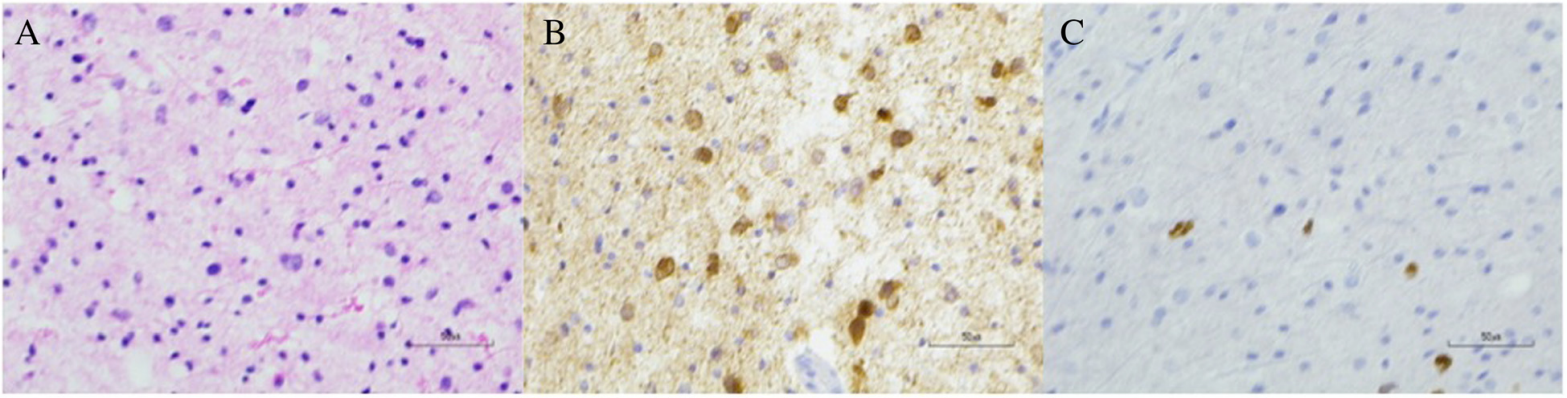
Histopathological features of the initial tumor. **a** A routine H&E stain demonstrated hypercellularity, nuclear atypia, and mitotic figures suggestive of anaplastic astrocytoma. **b** Immunohistochemical staining showed positivity for the IDH1 (R132H) mutation. **c** MIB-1 immunohistochemical staining showed a mitotic index of approximately 5 %

A 34-year-old male was incidentally found to have bilateral papilledema on a routine ophthalmological exam. His past medical history was significant for two episodes of generalized tonic-clonic seizures with negative work-up on imaging, for which he was on anti-convulsant medication. To further evaluate his papilledema, he was sent to the emergency department of a nearby hospital for further evaluation where a non-contrast CT showed a 3.7-cm hypo-dense mass in the right temporal lobe associated with 6 mm of leftward midline shift. A brain MRI re-demonstrated a non-enhancing lesion in the right temporal lobe (Fig. 1a) with significant peri-tumoral edema (Fig. 1b) that extended superiorly into the right fronto-parietal lobes and across the corpus callosum (Fig. 1c). There was no evidence of additional disease within the infra-tentorial compartment, including the cerebellum (Fig. 1d). Subsequently, the patient underwent subtotal resection of the temporal lesion with final pathology demonstrating a WHO III anaplastic astrocytoma (Fig. 2a) with intact 1p/19q chromosomal arms and positive for the IDH1 (R132H) mutation, based on immunohistochemical staining (Fig. 2b). The tumor exhibited a moderate mitotic index of 5 % (Fig. 2c).

After surgery, the patient was referred to the neuro-oncology branch of our institution for further management of his condition. Two months after the surgery, the whole tumor bed was radiated over 30 fractions to a total of 60 Gy but given the large volume of the radiation field, concurrent temozolomide was deferred to reduce risk of radiation-induced necrosis. Over the next year, he received 12 cycles of adjuvant temozolomide, which he tolerated well. Serial MRIs demonstrated attenuation of fluid-attenuated inversion recovery (FLAIR) signal abnormality, and clinically, the patient was doing very well and remained free of neurological symptoms.

**Fig. 3:**
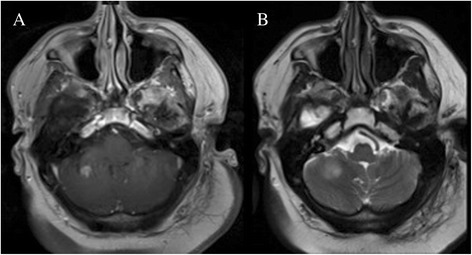
Imaging features of the recurrent tumor. **a** T1-weighted post-contrast and **b** T2-weighted MRI demonstrated an enhancing lesion in the right cerebellum with peri-lesional edema

**Fig. 4:**
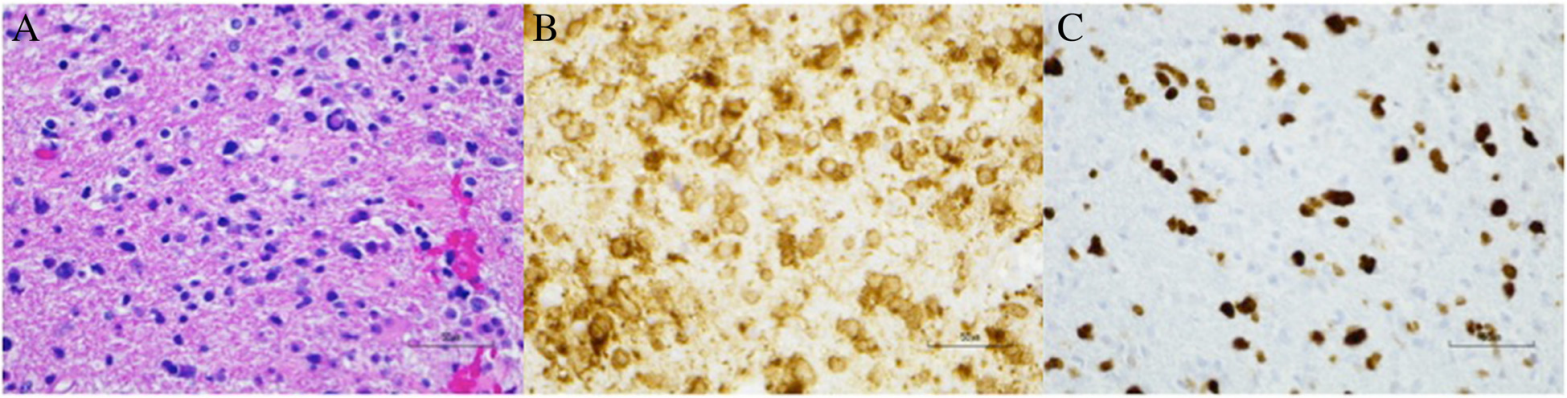
Histopathological features of the recurrent tumor. **a** A routine H&E stain showed histopathology similar to the initial tumor, including hypercellularity and anaplasia. **b** Immunohistochemical staining was again positive for the IDH1 (R132H) mutation. **c** MIB-1 immunohistochemical staining demonstrated a mitotic index of 40 %, which was significantly elevated in comparison to the initial lesion

Over the next year and a half, routine surveillance imaging obtained at approximate 6-month intervals showed local disease control and absence of distant recurrence. However, on an MRI taken 22 months after initial surgery, a new enhancing nodule was found in the right cerebellum (Fig. 3a) with local edema (Fig. 3b). The patient also co-incidentally described a 1-month history of imbalance particularly upon changing positions from sitting to standing. This lesion was felt to likely represent recurrent tumor although trans-tentorial spread would have been uncommon. Delayed radiation necrosis was also a possibility, given that upon review of radiation records, the affected area in the cerebellum had received 54–57 Gy. To guide in treatment decision-making, a definitive diagnosis was sought in the form of surgical resection of this lesion. Final pathological review of the specimen demonstrated an IDH1 (R132H)-mutated WHO III anaplastic astrocytoma (Fig. 4a, b). These findings were consistent with those from the patient’s first resection and were indicative of recurrent tumor. However, the mitotic index was markedly elevated at 40 % (Fig. 4c) compared to 5 % from the first surgery. As such, alternative chemotherapy was pursued, and the patient at the time of the reporting of this case was under treatment with procarbazine and CCNU.

### Discussion

Gliomas arising within the cerebellum are rare and reportedly make up 0.4–3.4 % of all gliomas, the majority of which present as grade IV glioblastoma [6]. However, cerebellar gliomas secondary to spread from a supratentorial tumor are exceedingly uncommon and, among previous case reports, have been limited to a single study by van Nifterik et al. [7]. The authors described a 31-year-old male who initially underwent subtotal resection of a left frontal glioblastoma multiforme (GBM), followed by external-beam radiotherapy (42 Gy in 3-Gy daily fractions) and single-fraction stereotactic boost (15 Gy) to the residual mass. Twenty months after surgery, he demonstrated tumor recurrence involving the center of the cerebellum, which was diagnosed as recurrent GBM after surgical resection. Genetic analyses of both tumors revealed identical loss of heterozygosity at TP53 along with absence of PTEN loss and EGFR amplification. IDH mutation status was not performed in this study given that the discovery of IDH mutations in gliomagenesis was published 3 years after this report. As such, our patient represents the second case of a supratentorial glioma recurring trans-tentorially and involving the cerebellum. Furthermore, unlike the report by van Nifterik et al. [7], our patient’s tumor exhibited WHO III grading, suggesting that trans-tentorial spread may not be a characteristic limited to the biology of WHO IV gliomas, alone.

It is unclear whether the improved prognosis associated with IDH mutations is due to an enhanced sensitivity to chemo- and radiotherapies or to less aggressive intrinsic biological behaviors. In support of the former possibility, two large-scale studies demonstrated that IDH mutations conferred higher response rates to temozolomide and combination procarbazine, lomustine, and vincristine therapy in low-grade gliomas and oligodendrogliomas, respectively [8, 9]. However, other studies in similar patient cohorts have failed to reproduce these findings [10, 11]. Likewise, in support of attenuated biology, IDH-mutated tumors may be more amenable to surgical resection [12], potentially due to favorable MRI characteristics, including reduced contrast enhancement, unilateral growth patterns, and sharper tumor margins [13]. When analyzing patterns of recurrence as a correlate of intrinsic biologic behavior, recent evidence also suggests that IDH mutations may predict a more benign clinical course. Recently, Shibahara et al. demonstrated that IDH wild-type WHO III anaplastic astrocytomas had significantly higher rates of distant recurrence along with increased expression of glioma stem cell markers compared to their IDH-mutated histological counterparts [14]. Out of 86 total patients, 15 exhibited distant recurrence, among which only 3 were IDH-mutated, none of which involved trans-tentorial spread like our patient. The authors concluded that IDH wild-type WHO III gliomas may behave more aggressively, more similarly to WHO IV glioblastomas. In regard to our patient, despite first-line treatment with temozolomide and radiation after initial tumor resection, our patient developed disease recurrence in a relatively short period of time and had distant recurrence. As such, the fact that our patient’s tumor was both treatment-refractory and behaved invasively suggests further factors contributed to its unusual clinical course. As no standard of care currently exists for initial treatment of WHO III astrocytic gliomas [15], we must consider the possibility that temozolomide treatment induced mutagenic changes, which led to heightened tumor aggression. This is supported by findings from Johnson et al., who demonstrated that treatment of low-grade gliomas with temozolomide could promote a hypermutated phenotype at recurrence and malignant progression into WHO IV glioblastoma [16]. Further investigation of our patient’s tumor is warranted to identify additional features that contributed to its unusual clinical course.

## Conclusions

Although debate continues over the underlying mechanism of increased survival observed in IDH-mutated gliomas, our patient demonstrates that IDH-mutated tumors are indeed capable of trans-tentorial spread and may not require WHO IV histopathological characteristics to manifest such invasive features. Regardless, the surgical approach for such diffusely infiltrating gliomas is challenging but continues to involve maximal surgical resection of areas involving contrast enhancement and FLAIR signal abnormalities [17]. For recurrent tumors, repeat cytoreductive surgery may confer additional survival for patients [18]. In rare cases of distant tumor recurrence, the role of surgery is less clear, but as our case demonstrates, surgery can help differentiate between tumor recurrence and radiation necrosis, which may subsequently affect treatment decision-making. Additional reports are needed to determine the optimal management of these patients and also further characterize patterns of distant recurrence in IDH-mutated gliomas.

## Consent

This study was approved under protocol 03-N-0164 by the Institutional Review Board of the National Institute of Neurological Disorders and Stroke at the National Institutes of Health. Written informed consent for publication of their clinical details and/or clinical images was obtained from the patient. A copy of the consent form is available for review by the Editor of this journal.
